# Differential protection by cell wall components of *Lactobacillus amylovorus* DSM 16698^T^against alterations of membrane barrier and NF-kB activation induced by enterotoxigenic F4^+^*Escherichia coli* on intestinal cells

**DOI:** 10.1186/s12866-016-0847-8

**Published:** 2016-09-29

**Authors:** Marianna Roselli, Alberto Finamore, Ulla Hynönen, Airi Palva, Elena Mengheri

**Affiliations:** 1 CREA-NUT, Consiglio per la Ricerca in Agricoltura e l’Analisi dell’Economia Agraria, Food and Nutrition Research Center, Via Ardeatina 546, 00178 Rome, Italy; 2 Department of Veterinary Biosciences, Division of Microbiology and Epidemiology, University of Helsinki, P.O. Box 66, 00014 Helsinki, Finland

**Keywords:** *L. amylovorus*, S-layer proteins, Cell wall, Membrane damage, NF-kB activation

## Abstract

**Background:**

The role of *Lactobacillus* cell wall components in the protection against pathogen infection in the gut is still largely unexplored. We have previously shown that *L. amylovorus* DSM 16698^T^ is able to reduce the enterotoxigenic F4^+^*Escherichia coli* (ETEC) adhesion and prevent the pathogen-induced membrane barrier disruption through the regulation of IL-10 and IL-8 expression in intestinal cells. We have also demonstrated that *L. amylovorus* DSM 16698^T^ protects host cells through the inhibition of NF-kB signaling. In the present study, we investigated the role of *L. amylovorus* DSM 16698^T^ cell wall components in the protection against F4^+^ETEC infection using the intestinal Caco-2 cell line.

**Methods:**

Purified cell wall fragments (CWF) from *L. amylovorus* DSM 16698^T^ were used either as such (uncoated, U-CWF) or coated with S-layer proteins (S-CWF). Differentiated Caco-2/TC7 cells on Transwell filters were infected with F4^+^ETEC, treated with S-CWF or U-CWF, co-treated with S-CWF or U-CWF and F4^+^ETEC for 2.5 h, or pre-treated with S-CWF or U-CWF for 1 h before F4^+^ETEC addition. Tight junction (TJ) and adherens junction (AJ) proteins were analyzed by immunofluorescence and Western blot. Membrane permeability was determined by phenol red passage. Phosphorylated p65-NF-kB was measured by Western blot.

**Results:**

We showed that both the pre-treatment with S-CWF and the co- treatment of S-CWF with the pathogen protected the cells from F4^+^ETEC induced TJ and AJ injury, increased membrane permeability and activation of NF-kB expression. Moreover, the U-CWF pre-treatment, but not the co-treatment with F4^+^ETEC, inhibited membrane damage and prevented NF-kB activation.

**Conclusions:**

The results indicate that the various components of *L. amylovorus* DSM 16698^T^ cell wall may counteract the damage caused by F4^+^ETEC through different mechanisms. S-layer proteins are essential for maintaining membrane barrier function and for mounting an anti-inflammatory response against F4^+^ETEC infection. U-CWF are not able to defend the cells when they are infected with F4^+^ETEC but may activate protective mechanisms before pathogen infection.

## Background

Lactobacilli, and especially *Lactobacillus* strains with probiotic features, are considered to confer several health effects to the host. In the gastrointestinal tract these bacteria may inhibit pathogen adhesion, modulate immune response and protect membrane barrier function from injurious agents and pathogens in both humans and animals [1–5]. It is well established that the disruption of gut membrane integrity allows pathogen entry through a leaky barrier and leads to the development of inflammatory reactions and intestinal diseases [6, 7]. The barrier function is maintained through the tight junctions (TJs), formed by a complex of proteins such as *zonula occludens* (ZO)-1, occludin and claudin, that link adjacent cells and seal the intercellular space [8]. Adherens junctions (AJs), consisting of the transmembrane protein E-cadherin, and intracellular catenin, contribute to cell-cell adhesion [9] and are required for TJ formation [10].

In pigs lactobacilli, especially *L. amylovorus* strains, are common members of the gut microbiota [11]. *Lactobacillus amylovorus* DSM 16698^T^, isolated from an unweaned piglet, is particularly abundant in piglets [12]. This strain has been shown to protect intestinal cells from membrane damage and inflammation induced by enteroxigenic *Escherichia coli* (ETEC) carrying F4 fimbriae [3, 13, 14]. F4^+^*E. coli* strains are major causative agents of enteric infections, diarrhoea and mortality in piglets [15].

The mechanisms underlying the probiotic health effects of lactobacilli are not fully understood. Recently, efforts have been put forward to understand the role of cell wall components in the probiotic effects. Surface (S)-layers are located at the outermost part of the cell wall of several *Lactobacillus* strains, including *L. amylovorus* DSM 16698^T^. They are composed of numerous identical protein subunits that form a symmetric and porous layer covering the entire bacterial surface. The S-layer protein (SLP) subunits are held together and connected to the underlying cell wall carbohydrates by non-covalent interactions. The subunits are poorly water-soluble and spontaneously form a layer, or precipitate, in aqueous solutions in vitro [16]. Some studies report that SLPs, isolated from different *Lactobacillus* species, mediate bacterial adherence or inhibit pathogen adhesion [17–20], counteract *Salmonella* invasion [21], protect cytoskeleton and membrane barrier from injury [22], and exert immunomodulatory activity [23, 24]. However, the results are insufficient or even controversial, which is partly due to the difficulty to obtain pure, soluble S-layer preparations [16, 25]. Hynönen et al. [25] have been able to prepare purified cell wall fragments (CWF) coated with recombinant *L. amylovorus* SLPs that allowed the presentation of the SLPs in a native symmetric organization resembling that present on the intact bacterial surface. They found that *L. amylovorus* SLPs only poorly adhered to intestinal cells, despite the adhesiveness of whole bacterial cells.

Other components of the bacterial cell wall, such as wall teichoic and lipoteichoic acids (WTA and LTA, respectively), have been shown to exert either pro- or anti-inflammatory activity. For instance, Kaji et al. [26] demonstrated that WTA and LTA revert IL-12 production, induced by certain *Lactobacillus* strains, towards IL-10 production in macrophages. Kim et al. [27] found that LTA from *L. plantarum* inhibited the pathogen-induced increase in platelet-activating factor receptor in human monocyte-like cells. In contrast, other authors have shown a pro-inflammatory role of *L. acidophilus* LTA in experimental colitis and polyposis in mice [28, 29].

Overall, there is a need to further elucidate the functions of *Lactobacillus* cell wall molecules and in particular to understand their role in the protection against pathogen infection in the gut. In our previous studies, we have shown that *L. amylovorus* DSM 16698^T^ prevented the F4^+^ETEC-induced disruption of TJ and cytoskeleton proteins in intestinal cells through IL-10-mediated signaling involving IL-8 down-regulation [13]. In addition, we have found that this strain inhibited the F4^+^ETEC-induced pro-inflammatory response by blocking the increase in NF-kB signaling [3]. In the present study, we have investigated the role of particular cell wall components of *L. amylovorus* DSM 16698^T^ in the protection against F4^+^ETEC-induced membrane barrier damage and inflammation. We used purified CWF as such, i.e. uncoated (U-CWF) or coated with SLPs (S-CWF) purified from *L. amylovorus* DSM 16698^T^, according to previous studies [25, 30]. As intestinal cells, we used the Caco-2 cell line, expressing several morphological and functional characteristics of mature enterocytes, such as a well-differentiated brush border on the apical surface and TJs [31].

## Methods

### Epithelial cell culture

The human intestinal Caco-2/TC7 cell line was kindly provided by Monique Rousset (Institute National de la Santé et de la Recherche Médicale, INSERM, France). These cells derive from parental Caco-2 cells at late passage, exhibit a more homogeneous expression of differentiation traits, and have been reported to express higher metabolic and transport activities than the original cell line, more closely resembling small intestinal enterocytes [32]. The cells were routinely maintained at 37 °C in an atmosphere of 5 % CO_2_/95 % air at 90 % relative humidity. The cells were used between passages 100 and 105 and routinely grown on plastic tissue culture flasks (75 cm^2^ growth area, Becton Dickinson, Milan, Italy) in Dulbecco’s modified minimum essential medium (DMEM; 3.7 g/L NaHCO_3_, 4 mM glutamine, 10 % heat inactivated fetal calf serum, 1 % nonessential amino acids, 10^5^ U/L penicillin and 100 mg/L streptomycin). All cell culture reagents were from Euroclone (Milan, Italy). For the experiments, the cells were seeded on Transwell filters (polyethylene terephtalate filter inserts for cell culture; Becton Dickinson) of 12 mm diameter, 0.45 μm pore size, as described below. After confluency, cells were left for 17–21 days to allow differentiation [31]. Medium was changed 3 times a week.

### Bacterial strains and culture conditions

The F4^+^ETEC (O149:K88ac; kindly provided by The Lombardy and Emilia Romagna Experimental Zootechnic Institute, Reggio Emilia, Italy) was grown in Luria-Bertani (LB) broth containing 1 % tryptone and 0.5 % yeast extract (both from OXOID, Basingstoke, England), plus 1 % NaCl, pH 7.0. After overnight incubation at 37 °C with vigorous shaking, the bacteria were diluted 1:200 in fresh LB and grown until mid-log phase. Bacterial cells were then harvested by centrifugation at 3000 × g for 10 min at 4 °C and resuspended in antibiotic- and serum-free DMEM.

*L. amylovorus* strain DSM 16698^T^_,_ isolated from piglet small intestine and previously called *L. sobrius*, (kindly provided by H. Smidt, University of Wageningen, Wageningen, The Netherlands) was grown in DeMan Rogosa Sharp (MRS) medium (DIFCO, Milan, Italy) at 37 °C under anaerobic conditions.

Bacterial concentrations were determined by densitometry and confirmed by serial dilutions followed by colony forming unit (CFU) counts of F4^+^ETEC on LB agar after 16 h incubation, and of *L. amylovorus* DSM 16698^T^ on MRS agar after 48 h incubation at 37 °C, under anaerobic conditions. The viability of F4^+^ETEC grown on DMEM did not differ from that of bacteria grown on LB medium, as tested in preliminary experiments by CFU counts after agar plating of bacterial inoculates from the two different media.

### *L. amylovorus* DSM 16698^T^ S-CWF and U-CWF preparations

*L. amylovorus* DSM 16698^T^ S-CWF and U-CWF were prepared as described by Hynönen et al. [25], except that SLPs isolated from the bacterial surface [30], rather than recombinant SLPs, were used. Briefly, SLPs were extracted from the bacterial surface by 5 M guanidine hydrochloride (GHCl) and purified by size exclusion chromatography in 5 M GHCl in a Sephacryl S-200 HiPrep 16/30 column, after which CWF, prepared as described by Avall-Jääskeläinen et al. [33], were coated with the purified SLPs. U-CWF were used as negative controls.

### Cell treatments

Caco-2/TC7 cells, differentiated on Transwell filters of 12 mm diameter (1 × 10^6^ cells/filter) were untreated (control, C), or apically treated with 1 mL of medium containing F4^+^ETEC (5 × 10^6^ CFU/mL; E), S-CWF (amount equivalent to 5 × 10^7^ CFU/mL of *L. amylovorus* DSM 16698^T^) or U-CWF (amount equivalent to 5 × 10^7^ CFU/mL of *L. amylovorus* DSM 16698^T^), or co-treated with S-CWF or U-CWF and F4^+^ETEC, for 2.5 h in a humidified atmosphere of 5 % CO_2_. Other cell monolayers were pre-treated with S-CWF or U-CWF for 1 h before F4^+^ETEC infection (prS-CWF + E or prU-CWF + E), as described above. We determined the pathogen concentration and time of incubation based on preliminary experiments to allow the triggering of the inflammation pathway without the disruption of the cell monolayer [3]. The 1:10 ratio of F4^+^ETEC to *L. amylovorus* DSM 16698^T^ was that used in our previous study [13].

### Localization of TJ (ZO-1 and occludin) and AJ (E-cadherin and β-catenin) proteins

Caco-2/TC7 cells were washed three times with cold PBS containing Ca^++^ and Mg^++^, fixed in ice-cold methanol for 3 min, and then incubated with rabbit polyclonal anti-ZO-1, mouse monoclonal anti-occludin, mouse monoclonal anti-β-catenin, or rabbit polyclonal anti-E-cadherin antibodies (Zymed Laboratories), for 1 h. For secondary detection, the cells were incubated with fluorescein isothiocyanate (FITC) or tetramethylrhodamine isothiocyanate (TRITC) conjugated secondary antibodies (Jackson Immunoresearch, Milan, Italy), for 1 h. Stained monolayers were mounted on glass slides by using Prolong Gold antifade Reagent (Molecular Probes, Invitrogen, Milan, Italy) and analyzed using a fluorescence microscope (Zeiss, Jena, Germany).

### Immunoblot analysis of TJ and AJ proteins

Cells were washed and lysed in cold radioimmunoprotein assay buffer (RIPA: 20 mM Tris-HCl pH 7.5, 150 mM NaCl, 0.1 % SDS, 1 % Na deoxycholate, 1 % Triton X-100) containing 1 mM phenylmethylsulphonyl fluoride, Protease Inhibitor Cocktail (Complete Mini, Roche, Milan, Italy) and Phosphatase Inhibitor Cocktail (PhosSTOP). The cells were centrifuged at 15,000 × g for 20 min and supernatants were recovered. Cell lysates (50 μg total proteins) were dissolved in sample buffer (50 mM Tris-HCl, pH 6.8, 2 % SDS, 10 % glycerol, 100 mg/mL bromophenol blue, 10 mM β-mercaptoethanol), heated for 5 min, fractionated by SDS polyacrylamide gel electrophoresis and transferred to nitrocellulose filters (Whatman Protran, PerkinElmer, Milan, Italy). The membranes were incubated with the following primary antibodies: mouse monoclonal anti-occludin (Zymed Laboratories, Milan, Italy), rabbit polyclonal anti-β-catenin, anti-phospho β-catenin, anti-E-cadherin and anti-α-tubulin (Cell Signaling Technology, Danvers, MA) at a concentration of 2 mg/L in 3 % BSA, for 1 h. The membranes were then incubated with horseradish peroxidase-conjugated secondary antibodies (Cell Signaling Technology), for 1 h. Proteins were detected using enhanced chemiluminescence reagent (ECL kit LiteAblot Extend, Euroclone), followed by analysis of chemiluminescence with the CCD camera detection system Las4000 Image Quant (GE Health Care Europe GmbH, Milan, Italy). Relative expression levels of occludin, β-catenin and E-cadherin proteins were normalized to α-tubulin, whereas the phosphorylated β-catenin was normalized to the corresponding unphosphorylated form.

#### Cell permeability

Cell permeability was measured by phenol red passage, according to Ferruzza et al. [34]. Briefly, following three washes of cell monolayers with PBS containing Ca^++^ and Mg^++^, 0.5 mL of 1 mM phenol red was added in the apical (AP) compartment, whereas 1 mL of PBS was added in the basolateral (BL) compartment. After 1 h of incubation at 37 °C, 0.9 mL of BL medium was collected, treated with 0.1 mL of 0.1 N NaOH and read at 560 nm to determine the phenol red concentration (Tecan Infinite M200 microplate reader, Tecan Italia, Milan, Italy). The phenol red passage was expressed as apparent permeability (P app), as previously described [34]. The TJs were considered open when the apparent permeability of phenol red was ≥ 1 × 10^−6^ cm x s^−1^.

#### Immunoblot analysis of NF-kB activation

The cells were processed as described above for immunoblot analysis of TJ and AJ proteins. After separation by SDS polyacrylamide gel electrophoresis and transfer to nitrocellulose filters, the membranes were incubated with rabbit polyclonal anti-p65 or anti-phospho(P)-p65 primary antibodies (Cell Signaling Technology, Danvers, MA) and then with horseradish peroxidase-conjugated secondary antibodies (Cell Signaling Technology). Chemiluminescence was analyzed as described above. The relative expression level of the phosphorylated protein was normalized to its corresponding unphosphorylated form.

#### Statistical analysis

The significance of the differences was evaluated by one-way ANOVA followed by *post-hoc* Tukey HSD test. The significance was set at *P* values < 0.05. The symbol * was used to indicate significant difference from the control group, whereas the symbol # was used to indicate significant difference from the ETEC group. All statistical analyses were performed with the software program “Statistica” (version 5.0; StatSoftInc, Tulsa, OK).

## Results

### Different protection of membrane barrier integrity by *L. amylovorus* DSM 16698^T^ S-CWF and U-CWF

To verify whether *L. amylovorus* SLPs and/or U-CFW were crucial for protection against the barrier damage induced by F4^+^ETEC, we analyzed the major TJ and AJ proteins, as well as membrane permeability in Caco-2/TC7 cells pre-treated with S-CWF or U-CWF and then infected with F4^+^ETEC, or co-treated with S-CWF or U-CWF and F4^+^ETEC.

#### Localization of ZO-1, occludin, E-cadherin and β-catenin

**Fig. 1:**
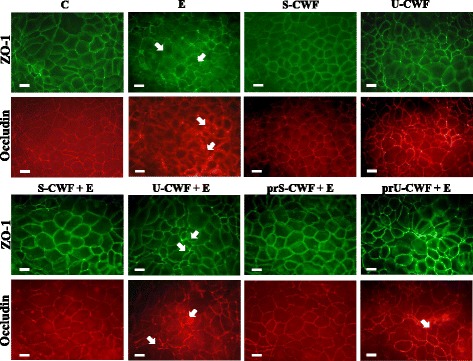
S-layer protein-coated or uncoated cell wall fragments differently protect tight junctions (TJs) in F4^+^ETEC infection. Caco-2/TC7 cells were: untreated (control, C), infected with ETEC (E), treated with S-layer protein-coated cell wall fragments (S-CWF) or uncoated cell wall fragments (U-CWF), co-treated with S-CWF or U-CWF and E (S-CWF + E or U-CWF + E, respectively), pre-treated with S-CWF or U-CWF before F4^+^ETEC infection (prS-CWF + E or prU-CWF + E, respectively). Cells were labelled with specific primary antibodies for ZO-1 and occludin, followed by FITC- and TRITC- conjugated secondary antibodies for ZO-1 and occludin, respectively*.* The figure shows the interruption of continuous staining of ZO-1 and occludin, resulting in dissociation of the proteins from membranes in F4^+^ETEC infected and U-CWF + F4^+^ETEC treated cells (arrows). Regular localization of TJ proteins is visible in cells pre-treated with S-CWF or co-incubated with S-CWF and F4^+^ETEC. A delocalization of occludin is present only in few cells pre-treated with U-CWF (arrow). Each figure is representative of three independent immunofluorescence assays (63 × magnification). Bars represent 10 μm

**Fig. 2:**
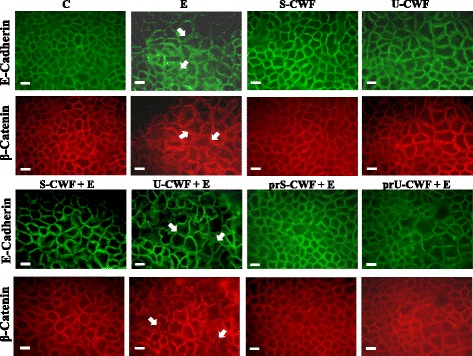
S-layer protein-coated or uncoated cell wall fragments differently protect adherens junctions from F4^+^ETEC-induced injury. Caco-2/TC7 cells were: untreated (control, C), infected with F4^+^ETEC (E), treated with S-layer protein-coated cell wall fragments (S-CWF) or uncoated cell wall fragments (U-CWF), co-treated with S-CWF or U-CWF and E (S-CWF + E or U-CWF + E, respectively), pre-treated with S-CWF or U-CWF before F4^+^ETEC infection (prS-CWF + E or prU-CWF + E, respectively). Cells were labelled with specific primary antibodies for E-cadherin and β-catenin, followed by FITC- and TRITC-conjugated secondary antibodies for E-cadherin and β-catenin, respectively. The figure shows the displacement of the proteins from cell-cell junctions in F4^+^ETEC infected and U-CWF + F4^+^ETEC treated cells (arrows). The regular cell-cell interactions is preserved in cells pre-treated with S-CWF or co-incubated with S-CWF and ETEC. An irregular staining of the proteins is present only in few cells pre-treated with U-CWF (arrows). Each figure is representative of three independent immunofluorescence assays (63 × magnification). Bars represent 10 μm

The immunolocalization of TJ proteins is shown in Fig. 1. The treatment of Caco-2/TC7 cells with either S-CWF or U-CWF alone did not modify ZO-1 and occludin localization around the cell boundaries. Infection with F4^+^ETEC caused TJ disruption, as indicated by ZO-1 and occludin delocalization from the membrane with scattered distribution of occludin inside the cells. The pre- and co-treatments of infected cells with S-CWF protected the cell membrane by maintaining the correct distribution and organization of TJ proteins. The membrane integrity was also preserved by the pre-treatment with U-CWF before F4^+^ETEC infection, however, a few cells showed occludin delocalization . On the other hand, the co-treatment with U-CWF was not able to counteract the membrane damage. The localization of E-cadherin and β-catenin (Fig. 2) was not affected by the treatment with S-CWF and U-CWF alone. F4^+^ETEC induced severe architectural disturbance of both proteins, as shown by irregular staining indicating displacement of the proteins from cell-cell junctions. In the presence of F4^+^ETEC, the S-CWF treatments counteracted all the alterations induced by this pathogen preserving the correct distribution and organization of the AJ proteins. On the contrary, the co-treatment with U-CWF and F4^+^ETEC did not preserve the AJ structure. However, when U-CWF were given to the cells before F4^+^ETEC infection, a correct AJ protein localization was found with the exception of few cells showing some delocalization of these proteins.

#### Immunoblot of occludin, β-catenin, P-β-catenin and E-cadherin

**Fig. 3:**
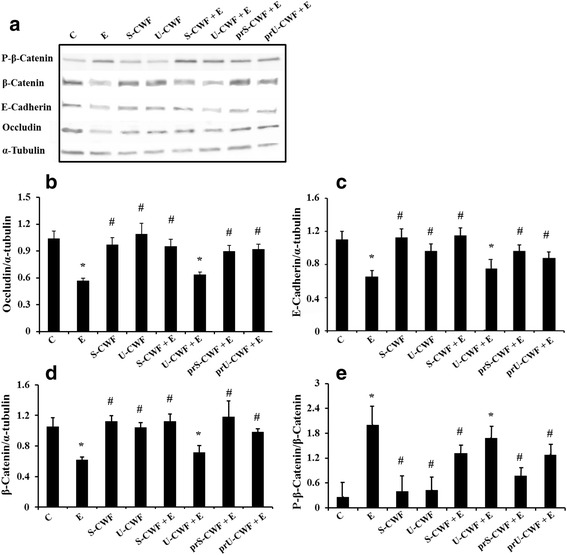
S-layer protein-coated or uncoated cell wall fragments dissimilarly counteract F4^+^ETEC-induced injury of membrane barrier. Caco-2/TC7 cells were untreated (control, C), infected with F4^+^ETEC (E), treated with S-layer protein-coated (S-CWF) or uncoated cell wall fragments (U-CWF), co-treated with S-CWF or U-CWF and E (S-CWF + E or U-CWF + E, respectively), pre-treated with S-CWF or U-CWF before F4^+^ETEC infection (prS-CWF + E or prU-CWF + E, respectively). Cell lysates were fractionated by SDS-PAGE and transferred to nitrocellulose filters. The membranes were incubated with mouse monoclonal anti-occludin and rabbit polyclonal anti-β-catenin, anti-phospho (P)-β-catenin, anti-E-cadherin, anti-α-tubulin primary antibodies, and then with horseradish peroxidase-conjugated secondary antibodies. The figure shows a representative Western blot of each protein (panel **a**) and the densitometric values of the immunoreactive protein bands (panels **b**–**e**). The relative expression levels of occludin, β-catenin and E-cadherin were normalized to α-tubulin, whereas the phosphorylated β-catenin was normalized to the corresponding unphosphorylated form. Values represent means ± SD of at least three independent experiments, carried out in triplicate. Statistically significant differences are shown and * indicates *P* < 0.01 from the control group, ^#^ indicates *P* < 0.05 from the ETEC group

To confirm the ETEC-induced alterations in TJ and AJ protein localization and the protection by *L. amylovorus*, we have analyzed the abundance of these proteins (Fig. 3). Panel A shows a representative Western blot of each protein. A strong decrease in the amount of occludin (panel B), β-catenin (panel D) and E-cadherin (panel C) was observed in the cells infected with ETEC compared with the control group. Both the pre- and co-treatments with S-CWF inhibited these alterations, since the amounts of occludin, β-catenin and E-cadherin of S-CWF-treated cells were significantly higher than those of ETEC-treated cells and did not differ from those of control cells (panels B-D). Protection was induced by U-CWF only when added before F4^+^ETEC infection but not when co-treated with F4^+^ETEC. In fact, a decrease in all the TJ and AJ proteins similar to that induced by ETEC was observed in the cells co-treated with U-CWF and ETEC, while the amounts of occludin, β-catenin and E-cadherin in the cells pretreated with U-CWF did not differ from those of control cells (panels B-D). We have also analyzed the phosphorylation of β-catenin (panel E), which in this form is known to dissociate from cell-cell contacts and accumulate in the cytoplasm [35]. F4^+^ETEC significantly increased the level of β-catenin phosphorylation compared with control cells. When the cells were pre-treated with S-CWF or U-CWF and co-treated with S-CWF and F4^+^ETEC the value of β-catenin phosphorylation did not differ from that of control cells. However, also in this case, U-CWF when co-treated with F4^+^ETEC was not able to inhibit the increase in β-catenin phosphorylation. The treatment of the cells with S-CWF or U-CWF alone did not induce any change in these proteins, as compared with control cells.

#### TJ permeability

**Fig. 4:**
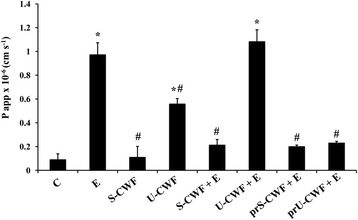
S-layer protein-coated- or uncoated-cell wall fragments protect against F4^+^ETEC-induced increase in membrane permeability differently. Caco-2/TC7 cells were untreated (control, C), infected with F4^+^ETEC (E), treated with S-layer protein-coated (S-CWF) or uncoated-cell wall fragments (U-CWF), co-treated with S-CWF or U-CWF and E (S-CWF + E or U-CWF + E, respectively), pre-treated with S-CWF or U-CWF before F4^+^ETEC infection (prS-CWF + E or prU-CWF + E, respectively). Phenol red was added in the apical (AP) compartment, for 1 h, and then determined spectrophotometrically in the basolateral (BL) compartment. The results are expressed as apparent permeability (P app). Values represent means ± SD of at least three independent experiments, carried out in triplicate. Statistically significant differences are shown and * indicates *P* < 0.001 from the control group, ^#^ indicates *P* < 0.001 from the ETEC group

Membrane barrier permeability was determined in Caco-2/TC7 cells by the paracellular flux of the phenol red marker (Fig. 4). The results were consistent with those of Western blot and immunofluorescence localization. In fact, F4^+^ETEC induced a significant increase in phenol red passage that was not observed after the pre- and co-treatments of infected cells with S-CWF. The pre-treatment, but not co-treatment with U-CWF, completely prevented the ETEC-induced phenol red passage increase. The treatment of the cells with S-CWF alone did not affect membrane permeability. In U-CWF treated cells, the phenol red passage was higher than that of control cells, but much lower than the reference value indicating an opening of TJ (1 × 10^−6^ cm x s^−1^).

### Inhibition of F4^+^ETEC-induced NF-kB activation by *L. amylovorus* DSM 16698^T^ S-CWF and U-CWF

**Fig. 5:**
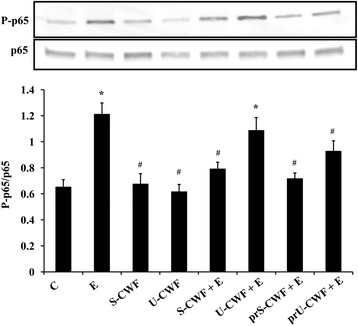
Inhibition of F4^+^ETEC-induced NF-kB activation by S-layer protein–coated or uncoated -cell wall fragments. Caco-2/TC7 cells were untreated (control, C), infected with ETEC (E), treated with S-CWF or U-CWF, co-treated with S-CWF or U-CWF and E (S-CWF + E or U-CWF + E, respectively), pre-treated with S-CWF or U-CWF before F4^+^ETEC infection (prS-CWF + E or prU-CWF + E, respectively). Cell lysates were fractionated by SDS-PAGE and transferred to nitrocellulose filters. Membranes were incubated with rabbit polyclonal anti-p65 or anti-phospho (P)-p65 primary antibodies and then with horseradish peroxidase-conjugated secondary antibodies. The figure shows the densitometric values of phospho (P)-p65 protein, normalized to its corresponding unphosphorylated form. Values represent means ± SD of three independent experiments, carried out in triplicate. Statistically significant differences are shown and * indicates *P* < 0.001 from the control group, ^#^ indicates *P* < 0.05 from the ETEC group

We further investigated whether the protection by *L. amylovorus* DSM 16698^T^ S-CWF and U-CWF was elicited through the inhibition of NF-kB signaling (Fig. 5). The infection of Caco-2/TC7 cells with F4^+^ETEC resulted in NF-kB activation, as shown by a significant increase in the level of the phosphorylated form of NF-kB subunit p65. This inflammatory response towards F4^+^ETEC was inhibited by S-CWF added either before (pre-treatment) or simultaneously (co-treatment) with the pathogen, as well as by the pre-treatment with U-CWF.

## Discussion

The disruption of intestinal barrier function may favor an indiscriminate passage of pathogens leading to an impairment of the mucosal immune system and the development of intestinal inflammatory diseases [6, 7]. In our previous study, we have shown that *L. amylovorus* DSM 16698^T^ reduces the adhesion of F4^+^ETEC and prevents the pathogen-induced disruption of TJ and cytoskeleton proteins through IL-10-mediated signaling involving downregulation of IL-8 [13]. We have recently also demonstrated that the same *L. amylovorus* DSM 16698^T^ strain inhibits the F4^+^ETEC-induced increase in NF-kB signaling [3]. In the present study, we show that SLPs isolated from *L. amylovorus* DSM 16698^T^, either in the pre-treatment before F4^+^ETEC infection or co-treatment with the pathogen, protected Caco-2/TC7 cells from the pathogen-induced TJ and AJ injury, increase in membrane permeability, and NF-kB activation. In other reports, a pre-treatment of T84 cells with SLP extracts from *L. helveticus,* before *E. coli* O157:H7 infection, was able to preserve membrane barrier function, while a co-treatment did not protect [18]. Interestingly, we also found that U-CWF were able to inhibit the membrane injury and NF-kB activation, but only if added to the cells before F4^+^ETEC infection. To our knowledge, this is the first report showing protection of intestinal cells from pathogen-induced injury by U-CWF. These results suggest that different components of *L. amylovorus* DSM 16698^T^ cell wall may counteract the damage caused by F4^+^ETEC and that different mechanisms are involved in the protection exerted by SLPs and U-CWF.

The functions of SLPs are still largely unknown. A possible mechanism explaining the protection against pathogens by SLPs is the competition for binding sites on intestinal cells, based on the ability of such proteins to mediate cell adhesion. For instance, the SLPs of *L. crispatus* inhibited adhesion of three different diarrhoeagenic *E. coli* strains (ETEC ATCC31705, enteroinvasive *E. coli* IID955 and enteropathogenic *E. coli* IID956) to the basement membrane through competition for binding sites with laminin molecules [19]. The pre-treatment of epithelial cells with SLPs from *L. helveticus* decreased enterohaemorrhagic *E. coli* adherence, attaching-effacing lesions and membrane barrier damage [18]. A reduced infection of *S. typhimurium* was associated with competitive exclusion in the gut and an enhanced immune protection conferred by the SLPs of *L. helveticus* in mice [20]. The ability of the SLPs from kefir-isolated *L. kefir* strains to co-aggregate with yeast *Saccharomyces lipolytica,* present in kefir grains [36], was associated with the inhibition of *Salmonella* invasion in intestinal cells [21]. However, the results of these studies are not fully conclusive, since SLPs in these preparations have not been purified and in soluble form [16]. Hynönen et al. [25] overcame this problem by a method based on the self-assembly of recombinant *L. amylovorus* DSM 16698^T^ SLPs on purified cell wall fragments to minimize SLP’s auto-precipitation and to allow the presentation of the proteins in the native organization, similar to that seen on the bacterial surface. These authors further studied the adhesive capacity of SLPs to porcine intestinal IPEC-1 cells by using SLP-CWF preparations with different SLPs isolated from several *L. amylovorus* strains. Surprisingly, they found a very low level of adhesion to intestinal cells by the SLPs of *L. amylovorus* DSM 16698^T^. This fact suggests that the protection elicited by S-CWF against F4^+^ETEC infection was not achieved through competition with the pathogen for binding sites. Although here Caco-2 instead of IPEC-1 cells were used, it is unlikely that S-CWF were able to induce a remarkable inhibition of F4^+^ETEC adhesion to Caco-2 cells. In fact, IPEC-1 and Caco-2 cells perform similarly in adhesion inhibition assays with intact *L. amylovorus* DSM 16698^T^ cells and F4^+^ETEC (data not shown). Moreover, even if a limited reduction of F4^+^ETEC attachment to Caco-2 cells had been induced by S-CWF, a sufficient number of pathogens would have adhered releasing toxins and causing membrane damage and inflammatory responses. Thus, beyond inhibition of adhesion, other mechanisms were involved in the protection elicited by S-CWF against F4^+^ETEC infection. This is in agreement with previous findings showing that an efficient inhibition of pathogen adhesion by different *Lactobacillus* strains is not necessary for the protection against pathogen-induced injury and that multiple mechanisms are responsible for the protective activities [3, 37–39].

In our previous studies, we have shown that *L. amylovorus* DSM 16698^T^ suppresses the activation of the different steps of TLR4 signaling in intestinal cells by inhibiting the F4^+^ETEC-induced increase of TLR4 and MyD88 levels, as well as the phosphorylation of the IKK-α, IKK-β, IkB-α and NF-kB subunit p65 [3]. These changes were associated with a decrease of inflammatory cytokines, including IL-8 that was involved in F4^+^ETEC-induced TJ injury [13]. In the present study, we found that *L. amylovorus* DSM 16698^T^ SLPs are essential to activate protective immune regulation in response to F4^+^ETEC infection, since the co-treatment of S-CWF with the pathogen inhibited NF-kB activation, while the co-treatment of U-CWF with F4^+^ETEC was ineffective. On the basis of previous and present results, we can suggest that the ability of isolated *L. amylovorus* DSM 16698^T^ SLPs to maintain membrane barrier structure and function is achieved through regulation of NF-kB mediated cytokine secretion.

The immunomodulatory importance of SLPs from different *Lactobacillus* species has also been demonstrated by previous studies. Konstantinov et al. [23] identified the SLPs of *L. acidophilus* NCFM as the binding ligand for dendritic cell-specific ICAM-3-grabbing non integrin (SIGN3), involved in the modulation of dendritic and T cells functions. Other authors have shown that the SLP purified from *L. acidophilus* NCK2187 binds to C-type lectin SIGNR3, which leads to a reduction of pro-inflammatory signals, mitigation of colitis and protection of gut barrier function in pathogenic T-cell-induced colitis in mice [40]. A modulation of innate immunity with inhibition of NF-kB activation was induced by the SLPs of *L. helveticus* MIMLh5 in Caco-2 cells, but not in human macrophages, where on the contrary, an increase of pro-inflammatory factors was found [24]. Johnson et al. [41] found reduced TNF-α production by dendritic cells when co-incubating them with a *L. acidophilus* NCFM LBA1029 deletion mutant lacking the Lba-1029 gene that encodes a putative S-layer associated protein, thus indicating that this SLP-associated protein is involved in the pro-inflammatory TNF-α response. Notably, in none of these studies a pathogen challenge was included. However, a recent study found an ability of the SLPs isolated from *L. acidophilus* to inhibit *Salmonella*-induced activation of MAPK signaling [22]. Consistent with this finding, our study highlights the role of SLPs in the gut protection against pathogen infection through the stimulation of innate immunity.

In our previous study, we have shown that TLR2 was required for the suppression of F4^+^ETEC induced TLR4 signaling activation by *L. amylovorus* DSM 16698^T^ [3]*.* Here, the fact that already a pre-treatment with U-CWF, prior to F4^+^ETEC infection, was able to block NF-kB activation, suggests that components of CWF may trigger protective mechanisms, including TLR2 activation, before F4^+^ETEC infection, which leads to NF-kB inhibition. LTA is a well-known and potent ligand of TLR2 [42]. As LTA was not present in the U-CWF preparation, we can speculate that WTA, similar in extracellular structure to LTA, had a role in TLR2 activation.

Previous studies have shown a pro-inflammatory role of LTA in intestinal inflammation. Mohamadzadeh et al. [28] found a down-regulation of IL-12 and TNF-α and amelioration of dextran sulphate-induced colitis in mice treated with *L. acidophilus* NCFM deficient in LTA. Other authors have found that oral treatment of mice with LTA-deficient *L. acidophilus* NCK2025 resulted in a reduction in inflammation and protection against colonic polyposis [29]. However, our results are in agreement with previous studies conducted in the presence of a pathogen, showing that LTA from *L. plantarum* exerted anti-inflammatory effects by inhibiting the NF-kB mediated increase in platelet-activating factor receptor induced by *Staphylococcus aureus* [27]. Moreover, WTA and LTA were identified as key factors for triggering the induction of IL-10 production mediated by TLR2-dependent ERK activation in macrophages [26].

## Conclusions

Our results indicate that the S-layer proteins from *L. amylovorus* DSM 16698^T^ are essential for maintaining membrane barrier function and mounting an anti-inflammatory response to F4^+^ETEC infection. Conversely, U-CWF are able to protect the cells through mechanisms exerted before pathogen infection.

In addition, this study supports the notion that not only viable probiotics induce health effects, but also their cell wall components or dead/inactivated bacteria induce beneficial immune and other biological responses, as recently reviewed [43–45]. Thus, our results suggest that the supplementation of *Lactobacillus* cell wall components may represent a therapeutic strategy to prevent or ameliorate infectious diseases in the gastrointestinal tract.

## Abbreviations

AJ: Adherens junction
CFU: Colony forming units
CWF: Cell wall fragments
ETEC: Enterotoxigenic *Escherichia coli*
LTA: Lipoteichoic acids
S-CWF: SLP-coated-CWF
SLP: Surface-layer protein
TJ: Tight junction
U-CWF: Uncoated-CWF
WTA: Wall teichoic acids
ZO-1: *Zonula occludens*-1
